# Proceedings of the Maryland and District of Columbia Dental Society

**Published:** 1880-02

**Authors:** 


AMERICAN JOURNAL

OF

DENTAL SCIENCE.

Vol. XIII. THIRD SERIES—FEBRUARY, 1880. No. 10

### ARTICLE I.

[Continued from last Number.]

*Proceedings of the Maryland and District of Columbia Dental Society. Annual Meeting.*

### FOOD AS A KINETIC.

"When a weight is lifted by the hand it seems a long way off to go to the sun for the muscular force employed in the act, yet the doctrine of the conservation of energy justifies the conclusion that its origin is there. (Pavy on Food, p. 20.) In 1843, Grove, of London, enunciated the doctrine of the 'Correlation of the Physical Forces' to wit: that force can neither be created nor destroyed. It may be variously combined and modified, but it remains the same in essence and unaltered in amount. The word 'correlation,' Grove indicated, meant, that any 'force capable of producing another, may, in its turn, be produced by it.'

In 1842, Mayer, of Germany, propounded the doctrine of the 'Conservation of force.' At about the same time, Mr.

Joule, of Manchester, England, discovered the equivalent of heat in mechanical motion. He found that heat enough to elevate *one pound* of water one degree Fahr., would, under another mode of action raise 772 pounds a foot high. So that one pound of water 772 feet high forms the dynamic (dunamos-force) equivalent of one degree of heat Fahr. Mr. Grove suggested that his doctrine could, in addition, be applied to the organic world, and that muscular force, animal and vegetable heat, &c. would be shown to possess similar relations. At this time organic processes were believed to be due to 'vitality' and the physical forces to be overruled by the 'vital principle.' Conceding that in the living organism there are influences that do not exist in dead matter, still, the effects may have their origin in the physical forces—the living matter forming the medium through which they operate. With artificial appliances force may be made to produce various effects, according to the nature of the instrument employed. With the same force in operation, different kinds of work are performed according to the character of the machine set in motion. Between the two—living matter and the machine—there exists an analogy which admits of being carried still further. It is only when in a certain state that matter is capable of forming the medium for the exercise of force in the production of living operations. Modify this state, and though there may be the same matter to deal with, yet it is no longer capable of fulfilling the same office it before performed: so in the case of an ordinary machine, it must possess a particular construction before it can form the medium for the operation of force. Disarrange this construction, and, although the matter remains unchanged, the application of force is without its proper effect. Thus a disarranged machine may be compared with living matter *devitalized.* In both, the capacity of being set in operation by force has existed, and in both that capacity has been lost. Further, it may be said that a machine in working order, but unoperated, is like matter possessing vitality but in a dormant state—both are ready to move directly the proper force is supplied." But how does the food furnish kinetic, (kineo—to move,) energy to muscles?

First, the force has its source in the food; if this is doubtful, let the doubtor stop eating entirely. Second, food comes primarily from the vegetable kingdom, animal food being derived from the vegetable. Third, except fungi and algae, vegetables grow through the influence of the sun's rays. "The energy contained in the rays are fixed or rendered latent in the vegetables." When an arrow-bow is bent the force derived from the muscular action employed in bending it is stored up, ready to be liberated when the trigger is pulled, and the force given to the arrow when it is launched is neither more nor less than that which has sprung from the muscular action employed in bending the bow. The same is true with vegetable products; their formation is coincident with the disengagement of oxygen from oxidized principles, and the development of combustible compounds. To effect this disengagement force is required. Now, the force so employed has its source in the heat and light evolved from the sun, and exists stored up in the product ready to be liberated on exposure to conditions favorable to oxidization. Our coal fields represent a vast magazine of potential force drawn ages ago from the sun's rays, and capable at any moment of being set free by the occurrence of oxidation. Plants are media for converting actual into latent or potential energy. Animals reconvert latent into various forms of actual force; hence, the analogy between a steam engine and the animal system—both convert latent into actual force. In the animal, food is the combustible material, and oxygen is supplied by respiration.

From the chemical energy due to the combination, force is liberated in the active state, and can perform work besides manifesting itself as heat and in other ways peculiar to the animal system. The steam engine is supplied with fuel which is the result of vegetation; it has the same respiration air as the animal, and from the combination in burning, heat is produced, some of which is lost, and some applied to mechanical work. Helmholtz says, the animal economy is more perfect than the steam engine in its capacity to turn force to account. It can utilize one-fifth of the power of its food, while the best steam engine utilizes only one-tenth—the rest escaping as heat. It is easy to see what an important food air is. We refer our readers to Pavy's admissible work itself. The scope of this part of our subject ranges into the most abstruse fields of science, showing how deep a problem food is.

*As a physiologic, or sustainer* of functions, or developer of organs. Of course, if all food is withheld, no function can be exercised after a time; but let us look at the functions—secretion and excretion—cerebration or nerve force—respiration—digestion—locomotion and circulation of the blood.

1. *Secretion.* This is a wonderful process. A cell that secretes milk possesses no visible difference from one that secretes as far as contents are concerned. The bile cell is more regularly polygonal than the milk cells. Both secretions are formed from the blood, yet the protoplasm (protos first, plasma mould or substance,) of the milk cell selects the material from the blood stream, and elaborates a liquid containing every element needful to make bone, muscle, sinew, nerves, cartilage, and every variety of tissue formed in the system, including the blood itself. Here is wrought an every day miracle on which the existence almost of our race depends! Theist and Atheist both agree that the protoplasm of the epithelial cells of the mammary gland is the active agent in this wonderful physiological chemistry. We that believe in God say, that here we are on the confines of the secret recesses of the Almighty, and that this process exemplifies the truth of Scripture, "For in Him we live and move and have our being." If not so, what, or who is the power acting behind and through the protoplasm?

We look out on a rich landscape in June, and note the herds of cattle clipping the luxuriant grass of the fields.

Later in the day we note their recumbent posture and rumination. Still later we see snow-white foaming milk as the product of the dairy. Now is not the transformation from the grass to the milk miraculous if it was not so common? We cannot imitate this phyto-chemistry in a laboratory. But how can we cause the animals to produce the best and most milk? Thousands of American dairymen have been at work answering this question, and it is solved. After allowing for differences in breeds and the individuals of these breeds, the interest of the milk producer centers upon the food. It must be ample, in good condition, served in a timely manner, and most important of all, it must be rich in all the chemical ingredients that are found in milk. There is room for much variation within certain limits, but fodder that is poor in mineral constituents will not answer. Hence, when drought diminishes the natural aliment of bovines, we find the dairymen giving out the tegumentary portion of grain that man rejects in making flour. Also they give excellent roots, rich in organic and inorganic constituents. They are carefully protected. When the natural aliment is thus supplied to healthy animals, experience proves that the satisfactory conditions have been realized.

The same general rule holds true of other secretions; they are all liable to vitiation unless the blood that supplies the respective glands contains the elements whence the individual secretion can be organized; so that while we are powerless to create a cell of ourselves, or to make it secrete milk or other liquid at will, we can limit or destroy or increase production by the way in which we feed. If this is so, is there not upon us a great responsibility? Does it not behoove parents and others to inquire as to these things, so that they may properly feed those in their charge?

2. *Cerebration or nerve food.* In point of fact the nervous system is paramount; without it, the bony, muscular, vascular, secretory, tegumentary and other systems are useless. In the nerves resides the man himself. We can suffer the loss of limbs, organs, but who can survive without nerves? Starve them and partial death ensues. As we can feel the bony system, so we can feel the nervous. Perhaps no idea in relation to food of late years has sunk deeper into the public mind than that attributed to Agassiz, to wit: that fish is good brain food. We have seen it denied that the professor ever made such a remark. It is strange if he did. See the analysis—(Pavy on Food, p. 171.)

Of white fish—whiting, haddock, cod, sole, turbot, brill, place, flounders, halibut, &c.

| | |
|---|---|
| Nitrogenous matter, | 18.1 |
| *Fat*, | 2.9 |
| Saline matter, | 1.0 |
| Water, | 78.0 |
| | 100.0 |

*Salmon.* (Pavy.)

| | |
|---|---|
| Nitrogenous matter, | 16.1 |
| Fat, | 5.5 |
| Salts, | 1.4 |
| Water, | 77.0 |
| | 100.0 |

Mackerel, herring, sprat and pilchard rank with salmon.

| | |
|---|---|
| *Eels.*—Nitrogenous matter, | 9.9 |
| Fat, | 13.8 |
| Salts, | 1.3 |
| Water, | 75.0 |
| | 100.0 |

Fat is counted as nerve food, but butter is all fat. Cheese is 24.1 per cent. fat. Cream, milk—eggs contain more than both. Besides, wheat, rye, barley, maize and oat containing the brick-like cells of gluten, rich in phosphates are better nerve food than fish. As we remarked, the æsthetic standard of whiteness requires the ablation of the gluten cells, because they make the bread dark colored. Now perhaps the price paid for adhering so closely to our standard of excellence without regarding other standards, may be more clearly understood when it is stated that in 1000 grains of wheat there are 8.2 grains of phosphoric acid ; this is equivalent to 150 doses of phosphorus when separated. 1000 grains of flour contain 2.1 grains of phosphorus, so that when a person wishes to take phosphorus in large quantity and in an assimilable form, one has only to eat wheat bread unbolted. Now, as the prevalence of insanity in modern times is somewhat alarming, and the best regimen for the feeble-minded and idiotic is to live on a diet that includes the normal amount of the mineral salts—is it too much to connect the excessive use of flour with affections of the nervous system? Fish is better than flour as a nerve food.

3. *Respiration.* Though there may be other fire places in the human body, the lungs are the chief seat of combustion by the union of food with the oxygen of the air respired. Also that sugar is the form in which food becomes lung fuel. The liver is a double gland, (Salisbury)—one forms bile, the other sugar. The portal veins carry the blood loaded with sugar from the liver to the lungs, where it is burnt up as fuel under a steam boiler. Both processes are not equally intense; one is slow and the other short, but the chemical results are all the same save in degree. Suppose no sugar or starch is taken as food; the liver will manufacture enough for the lung furnace. In case there is an excess of sugar after the normal utilization, it goes to form fat, and this state of things continuing, fat is deposited where it ought not to be. Hence sometimes we have Bright's disease, apoplexy, softening of the brain, and heart texture, &c. As things now are there is little danger of a lack of starch or sugar in the food—it lies in the opposite way.

4. *Circulation of the blood.* The main agent in this function is the impulse given by the heart's contractions. The blood runs with the velocity of a race-horse. Dr. Harriman estimates that if the arteries, veins and capillaries of one man could be put into one straight line, they would reach three times round our globe! The pressure of the blood is at least 2.5 lbs. to the square inch. The accomplishment of the circulation of the blood with such velocity and force, would be a miracle were it not for the 45,000,000 examples found in our nation. Just as the engines in the Holly system must have force enough to elevate the water and drive it through the pipes, just so must the heart have power sufficient to keep the life-current through the billions of blood-vessels. If the heart is weak, we find a lack of heat in the extremities, a pallor of the skin, generally combined with a state which, for the want of a better name, is termed "nervous prostration." This condition is well named, for give the heart plenty of nerve force and there will be less trouble with the circulation. So that the feeder must consult the neurotic aspect of food if an energetic circulation is required. Experience shows that the heart is well sustained by animal food which develops muscle and nerve. According to Dr. Salisbury, a slow beating heart is undesirable. It should contract he says, at least 72 times a minute and with force. Less rapid pulses are abnormal, while quicker pulses without fever is a sign of weakness; also the writer has found the best treatment for permanently cold feet, to consist in the use of animal food exclusively. Starch food has heat enough but does not confer nerve force nor make muscular fiber.

5. *Locomotion.* Assuming the bones to be in a normal state, we locomote by means of muscular force governed by nerve force. This division of our subject might have come under the last head. It includes pedestrianism, rowing, climbing, bicycling, ærostration when the force comes from the flyer, swimming, health lifting, jumping, skating, fighting, and any other motion by which man changes his position in space by his own efforts. Now oarsmen, prize fighters, and pedestrians have lately filled a large place in the public eye. The contestants pay strict attention to their food; they judge it not from the æsthetic view. We hope that the views of these classes of notorieties in relation to food will be regarded. It seems a small matter so far as any positive good is concerned, that A vanquishes B in a competition contest with feet, oars, or fists; but if the people learn from A's victory how to fit for locomotion, in the wide sense of the term, we can see that the national fame of a Hanlan or a Weston may be of more permanent value than the results we find recorded in the world's diary of events. The lesson is that *food* is a prime element in life. Cæsar's soldiers were conquerors because of the whole wheat food. British soldiers conquer because of their animal food rations. Dr. Livingston says, that the Makololo conquer because of animal food. Business men conquer because of their food. Porter-house steak lies at the foundation of more good bargains than doughnuts or hot flour biscuit. Feed Hanlan and Weston on cake and crackers and their laurels would soon be lost. "*Mens sana in sano corpore*," is what they need but they can't have them when feeding on impoverished food.

6. *Digestion*. A food that passes the æsthetic, chemic, and kinetic tests, and digests well is fit for royalty. If the object of food is vital, the repair of wasted tissues, the building up of the different systems, fuel, &c., then other things being equal, that aliment is the best which contains all the necessary elements, is easily, readily and mostly assimilable. On these grounds, animal takes the preference of vegetable. Muscles are easiest and soonest digested. What normally happens when a full meal is partaken, according to our present state of knowledge is as follows: generally, the fluid ingesta immediately enter the blood through the gastric walls by exosmose. Next we have the secondary digestion, circulating-function. By an unknown mysterious but probably simple process, the stomach is deluged to the amount of quarts, with a liquid, freely and quickly poured forth from the gastric walls. After a time, set by in some unknown power, this fluid is reabsorbed into the circulation of the blood, bearing the dissolved portions of the lean meat. Again, follows another pouring forth of the gastric deluge, the digestion of the lean meat, the reabsorption and so on. Starch, fats, and fibrous tissues are digested by the buccal, pancreatic, bile, and intestinal juices.

There are other processes, but the main point we would make, is that lean meat enters the blood in a shorter, quicker manner, and then it furnishes the most material to make muscle and nerve. In former times, strong differences of opinion have existed. Vegetarians have, and do now insist that no animal food should be eaten. We have no intention of engaging in the controversy, but would beg leave to state our belief in the Salisbury dictum, that two thirds animal and one-third vegetable food is a good combination for health.

The effect of cooking is to render the food more soluble. Starch has to become sugar or dextrine before it becomes soluble. If this is done by heat outside of the body, so much work is accomplished toward digestion. Iodine acts on cooked starch like uncooked. We have found that uncooked starch will polarize light, while cooked starch will not affect polarized light. This is seen in marked character in the ordinary bean; the starch grains are arranged into oval and ovoid bundles much like eggs in a basket, only the basket is continuous like a closely fitted envelope. The envelope is transparent, thick, tough, and made of cellulose. It resists the heat—hence the long time required to cook beans. After cooking, the outline of the grains is broken down and the envelope filled with a homogenous mass. When the cooking is not completed the peripheries of the starch grains are distinct and clear outlined and polarized light as aforesaid. Starch is called a colloid (glue like,) and not crystallizable. Its cousin, sugar, is a crystalloid, capable of crystallization. The digestion of the gluten and phosphate cells is not well understood. The cells that line the alimentary canal and secrete the digestive juices, have another function; that of absorption. They have an autonomy and select or *elect* (if you please,) the materials they choose. Why, and how this is so, is one of the riddles of protoplasm. The selected food is thus admitted to the lacteal and blood circulation, and carried over the body to supply waste of material, heat, force, &c.

It seems wisdom to ask in relation to food after it has passed the æsthetic tests, does it assimilate well, enter the circulations readily and with the least nerve force during the function we call digestion?

FOOD A POWER AS A CAUSE OF DISEASE OR PATHOLOGIC.

Dyspepsia (dys, difficult—pepsia, digestion,) depends on a sickness or disease of the eater or some defect in the food. The stomach and intestines may be functionally or organically diseased, or these may cause general condition of the body that incapacitates the stomach for digestion; but this is too purely a medical topic to be dwelt on here. We pass to the faults of food as pathologics. These arise from food being imperfect in itself or in cooking. It is a matter for congratulation that improper aliments usually betray themselves to the senses, and are rejected unless necessity force their use. For example, rotten potatoes, tainted meat, mouldy bread, soured milk, decayed eggs, &c. On the other hand, some food that is very agreeable acts like poison. Again some food is poisonous to one and not to another; this is not always explained. The dyspeptic often craves what experience shows will cause a diseased action. Lobsters, shell-fish, pork, veal, some kind of game, cheese, raw and unripe fruits, furnish labor for physicians. So also taenia, trichina, are found to come in meat, especially when eaten half cooked or raw. The advantage of cooking meat in relation to parasites, consists in coagulating the albumen of the parasites by 212° Fahr. In Abysinnia tape-worm prevails. This prevalence is supposed to be due to the habit the Abysinnians have of eating beef muscle raw and quivering from the live bovines standing at the door! But the question of interest is, can food such as is ordinarily employed produce disease?

In answer to this, Dr. Salisbury points out that strawberries eaten in excess have produced clots (thrombi,) in the blood, which, when caught in a blood-vessel and stopping it up are called "embol;" or plugs; this pathological state is termed *embolism.* The writer has verified this effect of strawberries. There are other causes of embolism, as the rheumatic diathesis, &c. Majendie fed dogs on flour solely—they died in the course of forty days with all the appearances of starvation. The corneas of the eyes ulcerated and the humors were evacuated, causing blindness. Hence the writer has queried if the use of flour too exclusively might not cause diseases of the organs of vision. On the other hand, Majendie found that dogs fed on wheat thrived. Food containing fat producing material may be expected to induce fatty degeneration of tissue as apoplexy, Bright's disease, weakened heart force. So tumors or abnormal enlargements in some instances are designated by Dr. Salisbury as diseases of nutrition, due to the excess of the carbohydrates in food; on the principle that a man who undertakes to raise potatoes, for example, in ordinary soil without manures, (soluble mineral food,) he would expect the vegetation to be ill-developed, and subject to organic diseases in the form of excresences and abnormal formation. Besides, he would find the plants infested with parasites that victimize their hosts.

As the outlook is now, it seems more than probable that a large variety of fatal diseases of man are due to parasites as a result of removing mineral salts from normal food. Beale has argued that because many parasites are harmless, therefore all are, and hence the idea of disease being caused by parasites as fungi and algae, is to him preposterous. It has not seemed to occur to this distinguished gentleman that the distinction of good and bad applies to all things of any class. As we have some phanerogamous plants, (trees, shrubs, &c.) noxious and some innoxious, so it is reasonable to suppose that cryptogams, (fungi, algae,) are subject to the same distinctions. In fact, many people have been reported as poisoned or dying from eating mushrooms. These simple facts show the fallacy of Beale's reasoning from analogy.

One of the most startling of modern diseases arises in that of Dr. Salisbury in relation to the causation, by feeding alone, of consumption. Were we at liberty to make full statements, it would show that he has demonstrated the synthesis of the most terrible scourge of the human race; also how to treat the disease in this principle. When his report is published, we think that this addition to our knowledge will be ranked as one of the foremost of the age. There is good reason to believe that cancerous affections are mainly due to feeding. Pathologists agree that cancer is a disease of *nutrition.* The tissues seem to be laid down under mob law; they riot without any governing or controlling force. In other words, the influences that keeps our bodies from being, for example, twenty feet in length, or our arms from being ten feet long or markedly symmetrically developed, is like the one that the condition we call cancer violates. The body system is like the body politic; it has laws that must be obeyed or else tissue nutrition ruin results. Now as food is the main factor in nutrition, the rest of the proposition is evident and comes under the next head.

### Food is a Power as a Therapeutic.

Organic disease is characterized by abnormal structural changes. What daily influence has more power over the body structures than food? It has to do with all. It goes to the intimate interstitial substance of every tissue. These change continually with great rapidity. Dr. G. B. Harriman, dentist, Boston, reports that a marked change from the chalky, soft, solid condition, to the firm, fibrous, tough texture of dentine has been observed by him in the space of three months. If such are the changes produced by feeding chemical food, how rapid must be the changes going on in the soft textures of the body! Indeed, the old idea that our bodies are renewed once in seven years, may yet be reduced to as many months. Now in cases of organic disease, by regulating the diet according to the general principles here laid down, the writer finds that abnormal growths which he had every reason to believe were malignant, have disappeared, and this in so many instances as not to be a mere coincidence.

The late Dr. Worthington Hooker was one of the pioneers in the reform of the diet of the sick. His idea was to give food liberally. He combated the prevalent notion that the sick need "light food." Starches, gruels, and jellies, certainly pass the æsthetic tests, but fail in the chemical; moreover, being mainly carbohydrates, they are apt to undergo saccharic acid fermentation in the alimentary canal, with the result of paralyzing the walls of the canal by the local action of carbonic acid gas. The peripheral cells lose the power of selective absorption, and take up materials not wanted. On the other hand, animal food is mainly digested in the stomach; not readily ferments, and is a good chemical food. It takes less nerve force to digest it. Its nourishing quality, bulk by bulk is many times more than the starches and sugars. If "light food" means an easily assimilable, small bulk, most nourishing aliment, animal food, must be termed "*light*"—popularly it has been thought "too hearty." This is a mistake, and thousands of graves have been filled by this doctrine. It is one of the best signs of the times that the sick get something to eat. The therapeutics of food never was better established than now.

In conclusion, we would say that we have endeavored to throw some light on food. We think that responsible people should not allow themselves to be solely influenced by the æsthetic view.

At the conclusion of the reading of the paper of Dr. Cutter, Dr. Coy moved that a vote of thanks be extended to Dr. Cutter. Carried.

The President appointed as Committee on Publication: Drs. H. M. Schooley, *Chairman ex-officio*; J. B. Hodgkin, C. E. Duck.

Drs. R. Finley Hunt, B. F. Coy, and T. S. Waters, were appointed as Committee on Scholarship.

On motion of Dr. Winder, it was agreed to read over Dr. Cutter's paper at our next meeting and discuss it.

The Association then adjourned *sine die*.

